# Sphingosine kinase-1 mediates endotoxemia-induced hyperinflammation in aged animals

**DOI:** 10.3892/mmr.2013.1562

**Published:** 2013-06-28

**Authors:** MARIA LUFRANO, ASHA JACOB, MIAN ZHOU, PING WANG

**Affiliations:** 1 Laboratory of Surgical Research, The Feinstein Institute for Medical Research, Manhasset, NY 11030, USA; 2 Department of Medicine, North Shore-LIJ School of Medicine, Manhasset, NY 11030, USA; 3 Department of Surgery, North Shore-LIJ School of Medicine, Manhasset, NY 11030, USA

**Keywords:** endotoxemia, aged, sphingosine kinase-1, hyperinflammation, sepsis

## Abstract

Sepsis is a serious issue in the geriatric population due to its association with high mortality rates in the elderly. The increase in mortality in the elderly correlates with inflammation. We have previously demonstrated that the inflammatory response is exacerbated in a rodent endotoxemia model of sepsis in aged rats compared with young rats. However, the molecular mediators associated with this hyperinflammatory response in aged rats have not been completely determined. Sphingosine kinase-1 (Sphk-1), an enzyme present in neutrophils and macrophages, regulates proinflammatory responses associated with endotoxemia and sepsis. To determine whether Sphk-1 is a molecular mediator associated with the observed hyperinflammatory response in aging, Sphk-1 mRNA expression was examined in hepatic tissues of young and aged rats subjected to endotoxemia. A significant increase in Sphk-1 mRNA was observed in endotoxemic aged rats compared with young rats. This increase was correlated with a significant increase in TNF-α mRNA levels in the liver. CD14 is a receptor component for lipopolysaccharide (LPS) and therefore, CD14 mRNA expression in hepatic tissues of endotoxemic young and aged rats was examined. Of note, CD14 mRNA was significantly upregulated in endotoxemic aged rats. Sphk-1 mRNA expression was significantly elevated in LPS-treated Kupffer cells and this increase correlated with an increase in CD14 mRNA expression. Results of the present study indicated that increased Sphk-1 expression in the liver in response to endotoxemia mediates the hyperinflammatory state observed in aged animals.

## Introduction

Sepsis, a systemic inflammatory response often resulting from an invasive bacterial infection, leads to multi-organ failure and mortality. In the United States, ~750,000 individuals develop sepsis each year (1,2). The vast majority of these individuals are at least 65 years old (3). The mechanism by which sepsis exerts its detrimental effects in the elderly has not been fully elucidated. A decline in immune function and an inadequate inflammatory response has been previously reported (4); however, the more accepted mechanism is an uncontrolled inflammatory response which, in turn, results in an excessive release of pro-inflammatory mediators and severe tissue injury. These observations are consistent with previous studies that have demonstrated a marked increase in the release of cytokines, particularly IL-6 and TNF-α, in association with more severe tissue injuries (5–7).

Sphingosine kinase (Sphk) is an intracellular signaling enzyme that catalyzes the phosphorylation of sphingosine to sphingosine-1-phosphate (S1P) (8). It is has been implicated in the regulation of immune cells, including neutrophils, monocytes and macrophages (9–11). Sphk-1 is emerging as an important mediator in inflammatory responses activated by various inflammatory stimuli, including lipopolysaccharide (LPS), TNF-α and IL-1β (12–15), and involves the toll-like receptor (TLR) signaling pathways. It has also been reported that Sphk-1 is upregulated in stimulated human phagocytes and peritoneal phagocytes of patients with severe sepsis and potentially plays a role in the development of sepsis (16). Blockade of Sphk-1 inhibits phagocyte production of endotoxin-induced pro-inflammatory cytokines, consistent with a study in which mice were pretreated with a Sphk-1 inhibitor following LPS administration, which revealed a decrease in TNF-α, IL-6, MCP-1 and HMGB1 (16). In addition, Sphk-1 and S1P have been demonstrated as important inflammatory mediators in asthma, rheumatoid arthritis and inflammatory bowel disease (IBD) (17–19). Sphk-1 expression has been found to increase in IBD and in a mouse model of colitis and Sphk-1 gene deficiency markedly decreased the systemic inflammatory response (20). Recently, expression of Sphk-1 and its activity were found to be markedly increased in peripheral immune cells of patients in the early stages of severe acute pancreatitis, indicating that the regulation of the Sphk-1 pathway may represent a novel target in the treatment of this disease (21).

Our previous studies demonstrated that levels of pro-inflammatory mediators were significantly elevated in aged animals following the induction of endotoxemia (6,7). Therefore, in the aged population, alterations in the immune response may contribute to a higher mortality rate following bacterial infection. The molecular mediators associated with this hyperinflammatory response are not clearly defined in the aged population. To determine whether Sphk-1 represents a molecular mediator associated with the observed hyperinflammatory response in aging, Sphk-1 mRNA expression was examined by quantitative PCR in hepatic tissues of young and aged rats subjected to endotoxemia, and its expression was found to correlate with CD14, a known component of the LPS receptor and the TLR signaling pathways.

## Materials and methods

### Experimental animals

Male Fischer-344 rats (young, 3 months old; aged, 24 months old) were obtained from the National Institute on Aging (Bethesda, MD, USA), housed in a temperature-controlled room on a 12-h light/dark cycle and fed a standard Purina rat chow diet. Experiments were performed in adherence with the National Institutes of Health Guidelines for the Use of Experimental Animals. This project was approved by the Institutional Animal Care and Use Committee of The Feinstein Institute for Medical Research (Manhasset, NY, USA).

### Cell culture and treatment

Kupffer cells were isolated separately from 6–8-week-old normal Sprague-Dawley rats by collagenase perfusion of the liver followed by Percoll gradient centrifugation as described previously (22). Kupffer cells were plated at 1×10^6^ cells/well in 6-well culture plates with DMEM containing 10% FBS and incubated overnight at 37°C. All media were supplemented with 10 mM HEPES (pH 7.4), 2 mM L-glutamine, 100 U/ml penicillin and 100 μg/ml streptomycin. Cells were incubated overnight in a 37°C incubator with 5% CO_2_. Cells were then washed with media and treated with 100 ng/ml LPS for 24 h.

### Induction of endotoxemia in rats

Endotoxemia was induced by intravenous administration of LPS, as described previously (6,7). Prior to the induction of severe endotoxemia, rats were fasted overnight, but allowed water *ad libitum*. Rats were then anesthetized with isoflurane inhalation, inguinal regions were shaved and washed with 10% povidone-iodine and a short subinguinal incision was made. The femoral vein was carefully separated from the artery and cannulated with a catheter (PE-50 tubing). A bolus injection of LPS (15 mg/kg; *E. coli* 055:B5 in 200 μl normal saline; Sigma-Aldrich, St. Louis, MO, USA) was administered through the femoral vein catheter. The same surgery was performed on the vehicle control animals, but the control was injected with normal saline instead of LPS. Tissue samples were collected at 4 h following LPS injection.

### Real-time PCR (Q-PCR) analysis

Sphk-1, TNF-α and CD14 gene expression was determined by Q-PCR. Total RNA was extracted from hepatic tissue using TRIzol reagent (Invitrogen Life Technologies, Carlsbad, CA, USA). Q-PCR was performed on cDNA samples reverse transcribed from 2 μg RNA using murine leukemia virus reverse transcriptase (Applied Biosystems, Foster City, CA, USA). Using a SYBR Green PCR Master mix (Applied Biosystems, Foster City, CA, USA), reactions were performed in a 24-μl final volume containing 0.08-μmol each forward and reverse primer, 2 μl cDNA, 9.2 μl H_2_O and 12 μl SYBR Green PCR Master mix. Amplification was performed using the Applied Biosystems 7300 Real-Time PCR machine under the thermal profile of 50°C for 2 min and 95°C for 10 min followed by 55 cycles at 95°C for 15 sec and 60°C for 1 min. Rat GAPDH mRNA expression was used to normalize each sample and analysis of each specific mRNA was conducted in duplicate. Relative expression of mRNA was calculated by the 2^−ΔΔCt^ method and results were expressed as fold change with respect to the corresponding experimental control. The rat primers used in the experiment are listed in Table I. To assess the specificity of the PCR products, a melting curve analysis was performed in each Q-PCR experiment. No non-specific products from any of the primers used in our experiments were detected.

### Measurement of TNF-α protein levels

Supernatants from Kupffer cells treated with LPS were measured for TNF-α protein levels using specific ELISA kits (BD Pharmingen, Franklin Lakes, NJ, USA).

### Statistical analysis

Data are expressed as the mean ± SEM and compared by one-way analysis of variance and the Student-Newman-Keuls Method for multiple groups and Student’s t-test for two groups. P<0.05 was considered to indicate a statistically significant difference.

## Results

### Sphk-1, TNF-α and CD14 mRNA expression in the liver of young and aged endotoxemic rats

**Figure 1:**
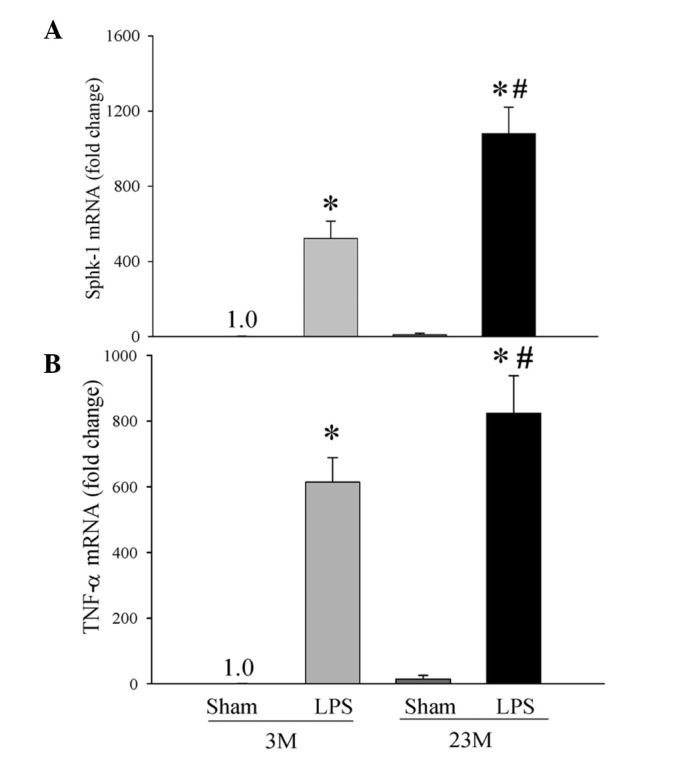
Sphk-1 and TNF-α mRNA expression in young (3 months old) and aged (23 months old) rats following endotoxemia. Total RNA was extracted from liver tissues of Sham and LPS-treated rats. (A) Sphk1 and (B) TNF-α mRNA expression was evaluated by Q-PCR and presented as fold change over GAPDH. Fold change in young Sham rats was set as 1.0. Data are presented as the mean ± SEM and compared by one-way ANOVA and Student-Newman-Keuls method. ^*^P<0.05, vs. Sham, ^#^P<0.05, vs. LPS 3M. Sphk-1, sphingosine kinase-1; LPS, lipopolysaccharide; Q-PCR, quantitative PCR.

We have previously demonstrated that the inflammatory response is exacerbated in a rodent endotoxemia model of sepsis in aged rats compared with young rats (6). To determine the mechanism by which the inflammatory response is exacerbated in aged rats in comparison with young rats, we examined gene expression of Sphk-1 in hepatic tissues of the young and aged animals during endotoxemia. As revealed in Fig. 1, gene expression of Sphk-1 in hepatic tissues was significantly increased following endotoxemia when compared with their respective Sham groups. There was a significant 2-fold increase in Sphk-1 mRNA expression in endotoxemic aged rats compared with that of young rats (1,080±141 vs. 522±92; P<0.001, Fig. 1A). The increase in Sphk-1 was correlated with a significant 1.3-fold increase in TNF-α gene expression during endotoxemia in the aged animals compared with young rats (824±113 vs. 614±74; P<0.05, Fig. 1B).

**Figure 2:**
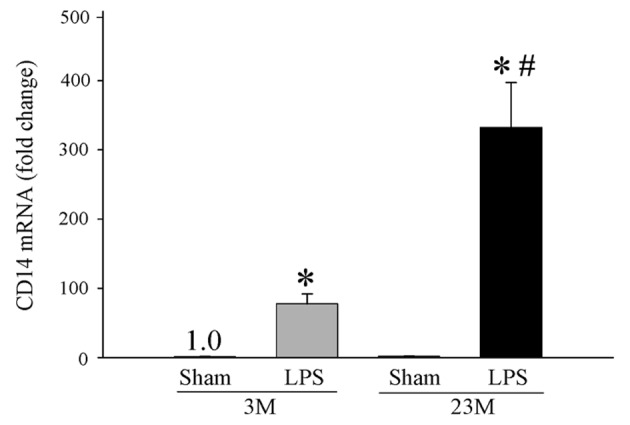
CD14 mRNA expression in young (3 months old) and aged (23 months old) rats following endotoxemia. Total RNA from liver tissues of Sham and LPS-treated rats was examined for CD14 mRNA expression by Q-PCR and presented as fold change over GAPDH. Fold change in young Sham rats was set as 1.0. Data are presented as the mean ± SEM and compared by one-way ANOVA and Student-Newman-Keuls method. ^*^P<0.05, vs. Sham, ^#^P<0.05, vs. LPS 3M. LPS, lipopolysaccharide; Q-PCR, quantitative PCR.

CD14, a known LPS receptor component, is important for the production of proinflammatory cytokines, including TNF-α, IL-1β and IL-6 (23,24). Gene expression of CD14 was significantly increased in young and aged endotoxemic rats compared with their respective Sham groups (Fig. 2). Of note, gene expression of CD14 was significantly increased by 4-fold during endotoxemia in the aged animals in comparison with the young animals (332±65 vs. 77±14; P<0.001, Fig. 2). These observations indicate a direct positive correlation among elevated Sphk-1, TNF-α and CD14 gene expression in hepatic tissues of endotoxemic aged rats.

### Sphk-1, CD14 and TNF-α mRNA expression in LPS-treated Kupffer cells

**Figure 3:**
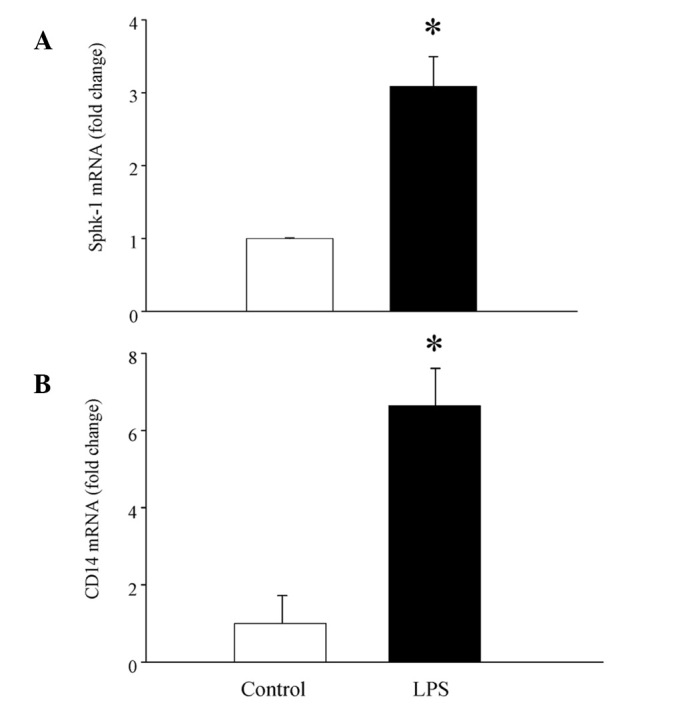
Sphk-1 and CD14 mRNA expression in LPS-treated Kupffer cells. Total RNA extracted from Kupffer cells treated with LPS was examined for (A) Sphk-1 and (B) CD14 mRNA expression by Q-PCR and presented as fold change over GAPDH. Data are presented as the mean ± SEM and compared by Student’s t-test. ^*^P<0.05 vs. control. Sphk-1, sphingosine kinase-1; LPS, lipopolysaccharide; Q-PCR, quantitative PCR.

To further delineate the signaling pathway responsible for the increased susceptibility of inflammation in the aged animals, mRNA from Kupffer cells was examined for Sphk-1, CD14 and TNF-α expression. Kupffer cells are specialized macrophages located in the liver. These cells line the walls of the sinusoids that form the reticuloendothelial system. Kupffer cells are a critical component of the innate immune system. As demonstrated in Fig. 3A, gene expression of Sphk-1 was increased by 3-fold while gene expression of CD14 was increased by 6.6-fold following treatment with LPS (Fig. 3B). TNF-α mRNA and protein expression was also significantly increased. These results indicate that increased expression of CD14 upregulates Sphk-1 in Kupffer cells and leads to increased TNF-α production, and thus mediates the hyperinflammatory response during endotoxemia in aging.

## Discussion

Sepsis is a major cause of morbidity and mortality in the geriatric population and ~60% of all sepsis cases occur in patients older than 65 years of age (3). Aging studies in human and animal models have revealed changes in a number of aspects of immunity (25–27). It has been hypothesized that the increased mortality rate of sepsis in the geriatric population is a direct result of an impaired immune response. We have previously reported that endotoxemia causes an *in vivo* hyperinflammatory state in aged animals (6,7). This was characterized by an increase in circulating and splenic levels of proinflammatory cytokines. A previous study demonstrated that Sphk-1 regulates proinflammatory responses associated with endotoxin and polymicrobial sepsis (16). In the current study, we focused on the potential role of Sphk-1 in the hyperinflammatory response observed in aged animals subjected to endotoxemia.

Sphk is an intracellular signaling enzyme that catalyzes the phosphorylation of sphingosine to S1P (8). A number of growth factors and cytokines have been demonstrated to activate Sphk, including platelet-derived growth factor, TNF-α and IL-1β (12,13,28). In the present study, the hepatic tissues of young and aged animals were examined to determine if Sphk-1 mRNA expression differed in these groups. LPS-treated aged animals were found to exhibit increased expression of Sphk-1 mRNA in hepatic tissues indicating a role for Sphk-1 in the hyperinflammatory response observed in the aged. Consistent with previous studies, increased Sphk-1 mRNA levels were also observed in LPS-treated animals in comparison with respective Sham groups. The exact signaling pathway by which Sphk-1 functions in association with inflammation has been studied by Puneet *et al* and has been hypothesized to involve TLR signaling. The authors found that LPS and bacterial lipoprotein markedly upregulates Sphk-1 expression. Sphk-1 acts through the second messenger, S1P, ultimately leading to the activation of NF-κB. NF-κB then leads to the release of TNF-α, IL-6 and IL-1β, and systemic inflammation (16). This previous study indicated a direct correlation between Sphk-1 and proinflammatory cytokines. Results of the present study are consistent with this proposed signaling pathway, whereby concomitant increases in Sphk-1, CD14 and TNF-α were identified in hepatic tissues of animals subjected to endotoxemia. Of note, increased levels of these proinflammatory mediators were found in the aged animal population. Thus, it appears that Sphk-1 may play a significant role in sepsis in the elderly by increasing the expression of proinflammatory cytokines.

To determine the cell type in the liver responsible for the observed increase in Sphk-1, we focused on Kupffer cells, which form part of the reticuloendothelial system and resident macrophages of the liver. These cells play a vital role in the innate immune response through the upregulation and release of proinflammatory cytokines (5). Increased Sphk-1 mRNA expression was identified in LPS-treated Kupffer cells compared with control samples. To further characterize the mechanism of action of Sphk-1, the expression of CD14 mRNA was studied in these cells. A statistically significant increase in expression of CD14 was found in LPS-treated cells. These results correlate with our *in vivo* observations with regards to a potential role of Sphk-1 in sepsis in the aged.

The role of CD14 as a key LPS signaling component has been well documented in a number of cell systems, including monocytes and macrophages (29–31). In the liver tissue, it has been previously reported that CD14 transcription rates significantly increased in the hepatocytes of LPS-treated rats (32,33). In addition, CD14 has been found to be expressed in an LPS-inducible manner in Kupffer cells and sinusoidal endothelial cells (34). Consistent with our observations, a previous study revealed that hepatic CD14 upregulation led to increased endotoxin sensitivity and host proinflammatory reactions, causing organ failure and mortality in a rat model of cholestasis (34,35). In addition, it has been hypothesized that the high levels of CD14 expression observed in Kupffer cells may increase proinflammatory responses and lead to increased endotoxin-induced mortality (36).

In summary, in the present study, increased expression of Sphk-1 was identified in young and aged animals subjected to endotoxemia compared with their respective Sham groups. Of note, the aged population was found to exhibit a significantly higher level of expression. This increased expression of Sphk-1 in the aged population corresponded with significantly higher levels of CD14 and TNF-α in the hepatic tissues. Increased expression of Sphk-1 and CD14 was also observed *in vitro* in Kupffer cells treated with LPS. While there was a 4-fold increase in CD14 expression, Sphk-1 only increased by 2-fold indicating that additional signaling components are likely to be involved in the hyperinflammatory state associated with endotoxemia in the aged. However, results of the current study collectively indicate that the hyperinflammatory state previously observed during endotoxemia in the aged may, in part, be due to the increase in Kupffer cell CD14 expression, leading to increased Sphk-1 and subsequent increases in TNF-α. Therefore, it is indicated that Sphk-1 contributes to age-related hyperinflammation in endotoxemia.

## Figures and Tables

**Table I:** Rat primers used in the present study.

Gene	Forward	Reverse
Sphk-1	TGCCTTCTCATTGGACTGTGG	GTAGCAGCACCAGCACCAG
CD14	TGGGCGAGAAAGGACTGATC	GGAGGGTCGGGAATTTGTG
TNF-α	TGATCGGTCCCAACAAGGA	GGGCCATGGAACTGATGAGA
GAPDH	ATGACTCTACCCACGGCAAG	CTGGAAGATGGTGATGGGTT

Sphk-1, sphingosine kinase-1.
